# Fat transplantation induces dermal adipose regeneration and reverses skin fibrosis through dedifferentiation and redifferentiation of adipocytes

**DOI:** 10.1186/s13287-022-03127-0

**Published:** 2022-10-09

**Authors:** Jing Wang, Junrong Cai, Qian Zhang, Jiaqing Wen, Yunjun Liao, Feng Lu

**Affiliations:** 1 Department of Plastic and Cosmetic Surgery, Nanfang Hospital, Southern Medical University, 1838 Guangzhou North Road, Guangzhou, 510515 Guangdong People’s Republic of China; 2 Department of Plastic Surgery, The Second Affiliated Hospital, Zhejiang University School of Medicine, Hangzhou, People’s Republic of China; 3 Department of Plastic and Reconstructive Surgery, Nanfang Hospital, Southern Medical University, Guangzhou, 510515 People’s Republic of China

**Keywords:** Scleroderma, Fat grafting, Adipocyte, Dedifferentiated adipocyte, Adipose-derived stem cell

## Abstract

**Background:**

Localized scleroderma causes cosmetic disfigurement, joint contractures, and other functional impairment, but no currently available medications can reverse the resulting skin lesions. Fat grafting is beneficial for reversing skin fibrosis; however, the mechanism by which adipose tissue transplantation contributes to lesion improvement has not been fully clarified. The purpose of our study was to verify the therapeutic effect of fat grafts in reversing skin fibrosis.

**Methods:**

Inguinal fat pads from AdipoqCreER+;mT/mG mice, which were treated with tamoxifen, were transplanted to the skin lesion in bleomycin-treated wild-type C57 mice. Tdtomato transgenic mice-derived adipocytes, adipose-derived stem cells (ASCs), dedifferentiated adipocytes (DAs) were embedded in matrigel and transplanted beneath the skin lesion of bleomycin-treated wild-type C57 mice. A transwell co‐culture system was used to verify the effect of ASCs, adipocytes or DAs on scleroderma fibroblasts or monocytes.

**Results:**

Adipocytes from the fat grafts could undergo dedifferentiation and redifferentiation for dermal adipose tissue re-accumulation within the skin lesion. Moreover, compared with ASCs and adipocytes, DAs show greater potency of inducing adipogenesis. ASCs and DAs showed comparable effect on inducing angiogenesis and suppressing macrophage infiltration in fibrotic skin. Co-culture assay showed that DAs and ASCs were able to reduce fibrosis-related genes in human scleroderma fibroblasts and drive M2 macrophage polarization.

**Conclusion:**

Our results indicated that adipocytes would transform into a more functional and dedifferentiated state and reverse dermal fibrosis, by promoting dermal adipose tissue regeneration, improving angiogenesis, suppressing macrophage-mediated inflammation and myofibroblast accumulation.

## Introduction

Scleroderma is a chronic autoimmune disease that results in skin and organ fibrosis and microvascular abnormalities [1–3]. Skin fibrosis occurs when myofibroblasts regarded as the primary fibrogenic effector cells in scleroderma, drive the excessive accumulation of fibrillar collagens and other extracellular matrix (ECM) proteins in the dermis [4, 5]. Subjacent dermal white adipose tissue (dWAT) and subcutaneous WAT (sWAT) are also affected in this condition. Loss of WAT accounts for the depression of the affected skin and characteristic skin tethering [6].

Previous studies have shown that attrition of the WAT layer in transgenic mice expressing ß-catenin in PDGRα+ progenitor cells result in a corresponding increase in dermal fibrosis [7]. Attrition of WAT and its replacement by fibrotic tissue in transgenic mice expressing Wnt10b in FABP4+ progenitor cells indicated that suppression of adipogenesis by Wnt–ß-catenin promotes emergence of fibrotic fibroblasts and replacement of dermal adipose tissue with fibrotic tissue [8]. Thus, WAT is closely associated with skin fibrosis; supplementation of subcutaneous adipose tissue is a potential alternative method for improving skin fibrosis in patients with scleroderma. Indeed, many clinical studies have reported autologous fat grafting as a treatment for hand, perioral, and other facial sclerotic changes in patients with scleroderma, while topical and oral treatments are unable to reverse skin fibrosis [9–12]. Several animal studies have also demonstrated that fat grafts exert an anti-fibrotic effect by reducing the numbers of macrophages and myofibroblasts in the skin lesion; thus, reversing the fibrotic phenotype [13, 14]. However, the mechanism by which fat grafts (or a cellular component in the fat grafts) contribute to anti-fibrotic effects remains unclear.

It has been reported that adipose-derived stem cells (ASCs) have potent immunoregulatory effects and can inhibit activation of macrophages and mitigate inflammation [15, 16]. Several studies have reported the use ASCs in the treatment of skin fibrosis and achieved satisfactory results [17–19]. However, few studies have focused on the therapeutic potential of adipocytes in reversing skin fibrosis.

Adipocytes are a group of cells that respond to various stimuli and have high plasticity. It has been reported that mature dermal adipocytes undergo dedifferentiation and redifferentiation under different physiological and pathophysiological conditions [20]. Following wounding, dermal adipocytes alter their fate and trans-differentiate into wounds using an unusual adhesion-independent, actomyosin-driven, peristaltic mode of motility to participate in the wound healing process [21, 22]. However, whether the adipocytes in transplanted fat tissues retain their plasticity and participate in the regeneration process of scleroderma skin lesions is unknown. In this study, our data reveal that adipocytes in transplanted fat tissue undergo dedifferentiation and redifferentiate into adipocytes to exert an anti-fibrotic effect.

## Materials and methods

### Animals

All experiments were approved by the Nanfang Hospital Animal Ethics Committee and were conducted in accordance with the guidelines of the National Health and Medical Research Council of China. C57/BL6 mice were obtained from the Southern Medical University (Guangzhou, China). B6.129-Tg (Adipoq-cre/Esr1*) 1Evdr/J (AdipoqCreER) and B6.129 (Cg)—Gt (ROSA) 26Sortm4 (ACTB-Tdtomato,-EGFP) Luo/J (mT/mG) mice (AdipoqCreERT2;mT/mG mice) were obtained from the Model Organisms Center, Inc. (Shanghai, China). Mice were maintained through routine breeding in the Laboratory Animal Center at Southern Medical University. Both male and female mice were used in these studies as no significant differences observed between the sexes. Animals were maintained on a standard chow diet ad libitum in a 12-h light/dark cycle. Two or three mice were housed per cage. All experimental procedures were approved and in accordance with the Institutional Animal Care and Use Committee.

### Induction of sclerosis via bleomycin treatment

C57/BL6 mice were administered bleomycin (BLM) to induce skin fibrosis. Briefly, BLM was dissolved in 0.9% phosphate-buffered saline (PBS) to make up the final concentration to 300 μg/μL. BLM (100 μL, 30 μg) was injected subcutaneously into both flanks of mice using a 27 G needle daily for 4 consecutive weeks. PBS (100 μL) was injected to the other group of mice as control.

### Mouse fat transplantation model

Seven days prior to fat harvest and transplantation, 100 μL of 30 μg/μL tamoxifen (Sigma, St Louis, USA) in sesame oil was intraperitoneally administered in AdipoqCreERT2;mT/mG mice to activate Cre activity. After activation, adipocytes which expressed adiponectin would turn into green fluorescence protein (GFP) -positive, while the other cells remained Tdtomato-positive. In this way, linear tracing of adipocytes could be conducted. The AdipoqCreERT2;mT/mG mice were anesthetized with isoflurane (1–3%) inhalation anesthesia. Fat tissue was harvested from the inguinal fat pads of mice and gently dissected into small pieces. Next, 200 μL of the prepared adipose tissue from AdipoqCreERT2;mT/mG mice or PBS were injected under the skin of the lower back of BLM-treated C57/BL6 mice using 16 G cannula. Skin lesions and fat grafts were harvested at 2 and 4 weeks after fat transplantation.

### Isolation and culture of adipocytes, DAs, and ASCs

Mouse adipocytes, dedifferentiated adipocytes (DAs) and ASCs were used for transplantation and co-culture assay with mouse monocytes. To obtain these cells, the epididymal fat pads of Tdtomato transgenic mice were minced into smaller pieces. Human adipocytes, DAs and ASCs were used for co-culture assay with human scleroderma fibroblasts. Human adipose tissue was harvested from abdomen or thigh of scleroderma patients by standard liposuction procedures with informed consent. The minced mouse fat tissue or human lipoaspirates were then treated with 0.2% type I collagenase (GIBCO, Invitrogen) at 37 °C for 35–40 min on a constant shaker at 200 rpm. Collagenase activity was terminated by the addition of an equal volume of complete medium consisting of low glucose Dulbecco’s modified Eagle’s medium (GIBCO), 10% fetal bovine serum (GIBCO), and 1% penicillin–streptomycin solution (Beyotime Biotechnology, Shanghai, China), and the cell suspension was centrifuged for 3 min at 1,000 rpm. The floating top layer containing unilocular adipocytes was harvested for subsequent adipocyte and DA culture. The bottom layer containing SVF was also collected for ASC culture.

To culture DAs, the obtained adipocytes were seeded at a density of 0.5–1 × 10^6^ cells per 25 cm^2^ flask. The flasks were fully filled with complete culture medium, which ensured that the floating cells were able to attach to the inner upper surface of the flask, and the cells were incubated at 37 °C in 5% CO_2_; this is termed as ceiling culture. After 48 h, the flasks were inverted. The medium was changed every 3 days until the cells reached confluence. The cells between passages 3 and 5 were used for further experiments.

To culture ASCs, the SVF was cultured on plastic culture dishes using Dulbecco’s modified Eagle’s medium supplemented with 2 mM L-glutamine, 100 U penicillin/100 U streptomycin, and 10% fetal bovine serum and sub-cultured when the cells reached 70–80% confluence. The ASCs cultured in passages 3 were used for further experiments.

### Injection of adipocytes, ASCs, and DAs to BLM-induced sclerosis

The BLM-treated C57/BL6 mice were anesthetized with isoflurane (1–3%) inhalation anesthesia and treated with Tdtomato transgenic mice-derived adipocytes (5 × 10^5^ cells embedded in 200 μL Matrigel), ASCs (5 × 10^5^ cells embedded in 200 μL Matrigel), DAs (5 × 10^5^ cells embedded in 200 μL Matrigel), or Matrigel (200 μL) beneath the skin lesion on the dorsal sides of mice using 16G needles. After 2 and 4 weeks, the mice were sacrificed and skin lesions were harvested.

### Histologic analysis

Skin lesions were fixed in paraformaldehyde for at least 48 h and embedded in paraffin. Sections (5 µm) were dewaxed using graded xylene and ethanol. Hematoxylin and eosin (H&E) as well as Masson’s trichrome (MT) staining was conducted according to the manufacturer’s instructions. CD31 (1:500; Abcam, Cambridge, UK), alpha smooth muscle actin (α-SMA, 1:200; Abcam) and perilipin A (1:800; Abcam) were used for immunohistochemical assays. For immunofluorescence assay, sections were incubated with following antibodies: F4/80 (1:100; Abcam), GFP (1:250; Abcam), Tdtomato (1:250; Abcam), Sox9 (1:200; Abcam) and then goat anti-chicken secondary antibody, Alexa Fluor 488 (1:500; Thermo Fisher Scientific, MA, USA) and goat anti-rabbit secondary antibody, Alexa Fluor 555 (1:500; Thermo Fisher Scientific). The cell viability was tested according to Calcein/PI staining kit (Beyotime Biotechnology, Shanghai, China). Images were obtained using an Olympus BX51 microscope (Olympus Corporation, Tokyo, Japan) or fluorescence microscope (Imager Z2, Carl Zeiss) and analyzed using the ImageJ software. Number of hair follicles and sebaceous glands was count manually on the samples with H&E staining.

### Harvest and culture of fibroblasts from scleroderma patients

Excised human scleroderma lesion samples were obtained from the patients who received lesion resection with informed consent. For human scleroderma fibroblast (SF) isolation, the tissue samples were washed with PBS twice and cut into 1 × 1 × 1 mm^3^ fragments followed by enzyme digestion with 0.2% collagenase type I (Sigma, St Louis, USA) dissolved in DMEM containing 10% FBS at 37 °C for 4 h with vigorous shaking. After centrifugation, the pellet was resuspended in DMEM containing 10% FBS, penicillin (100 U/mL), and streptomycin (0.1 mg/mL). Then, cells were seeded onto 10 cm culture dishes at a density of 1 × 10^4^ cells/cm^2^ and incubated at 37 °C in a humidified atmosphere staining 95% air and 5% CO_2_. The cells were passaged once 80–90% of confluency reached and then subcultured at the same density for two more passages. Passage three cells were harvested for the use of all experiments.

### Monocyte isolation

Murine bone marrow-derived macrophages (BMDM) were isolated from the bone marrow cells from mouse femurs. Then, the cell suspensions were filtered through a 100-micron nylon cell strainer, collected by centrifugation at 300*g* for 10 min, and resuspended in DMEM containing 10% FBS, 1% penicillin/streptomycin and GM-CSF (40 ng/mL, Thermo Fisher Scientific, Waltham, MA, USA). BMDMs were cultured at 37 °C with 5% CO_2_ for 7 days before all experiments. Recombinant LPS (100 ng/mL, Sigma, St Louis, USA) and recombinant GM-CSF (40 ng/mL) were used in preparing macrophage M1 inducing medium. IL-4 (10 ng/mL, Thermo Fisher Scientific) and recombinant GM-CSF (40 ng/mL) was used in preparing macrophage M2 inducing medium.

### Co-culture assays

To verify the effect of ASCs, adipocytes and DAs on SFs, a 6‐well transwell culture dish (Corning, USA) was used to develop the co‐culture system. Human ASCs, DAs and adipocytes were, respectively, inoculated on the upper chamber of the transwell culture dishes at a density of 1 × 10^4^ cells/cm^2^. Human SFs were inoculated on the lower chamber with the same density. The co‐cultured cells were set as an experimental group. As a control group, human SFs were inoculated on the lower chamber of 6‐well culture dishes at a density of 1 × 10^4^ cells/cm^2^ in DMEM plus 10% FBS and antibiotics. Co-cultures were performed over a total period of 48 h. After co-cultivation, SFs were collected for subsequently RT-qPCR experiments.

To test the effect of ASCs, adipocytes and DAs on BMDM, a 6‐well transwell culture dish (Corning, USA) was used to develop the co‐culture system. Mouse ASCs, DAs and adipocytes were, respectively, inoculated on the upper chamber of the transwell culture dishes at a density of 1 × 10^4^ cells/cm^2^. Mouse BMDMs were seeded on the bottom of the chamber with the same density. Co-cultures were performed over a total period of 48 h. After co-cultivation, BMDMs were collected for subsequently RT-qPCR experiments.

### Quantitative reverse transcription-polymerase chain reaction (qRT-PCR)

Total RNA was extracted using an RNeasy Lipid Tissue Mini Kit (Qiagen, Hilden, Germany) according to the manufacturer’s instructions. cDNA was synthesized using a QuantiTect Reverse Transcription Kit (Qiagen) and amplified for 40 cycles using a Rotor-Gene 3000 Real-Time PCR Detection System (Corbett Research, Sydney, Australia). GAPDH was used as the reference gene. Expression levels were calculated using the 2-ΔΔCt method. GAPDH was used as internal control. Primers used for qPCR are as follows: inducible nitric oxide synthase (iNOS) forward (5′-CCAACCTGCAGGTCTTCGATG-3′), iNOS reverse (5′-GTCGATGCACAACTGGGTGAAC-3′), tumor necrosis factor-α(TNF-α) forward (5′-CTCAAGCCCTGGTATGAGCC-3′), TNF-α reverse (5′-GGCTGGGTAGAGAACGGATG-3′), interleukin 6 (IL-6) forward (5′-TCTCCACAAGCGCCTTGG-3′), IL-6 reverse (5′-CTCAGGGCTGAGATGCCC-3′), Arg-1 forward (5′-CCTGAAGGAACTGAAAGGAAAGTT-3′), Arg-1 reverse (5′-GCAAGCCGATGTACACGATGT-3′), CD206 forward (5′-CTCTAAGCGCCATCTCCGTT-3′), CD206 reverse (5′-ATGATCTGCGACTCCGACAC-3′), interleukin-10 (IL-10) forward (5′-ATCGAACAAGGTATGCGAGG-3′), IL-10 reverse (5′-CTGGGGACAGTTCTGGGTTC-3′), collagen I forward (5′-GGCGGCCAGGGCTCCGACCC-3′), collagen I reverse (5′-AATTCCTGGTCTGGGGCACC-3′), Collagen III forward (5′-CAACCAGTGCAAGTGACCAA-3′), collagen III reverse (5′-GCACCATTGAGACATTTTGAAG-3′), α‐SMA forward (5′-CCCAGACATCAGGGAGTAATGG-3′), α‐SMA reverse (5′-TCTATCGGATACTTCAGCGTCA-3′), fibronectin forward (5′-GCCACTGGAGTCTTTACCACA-3′), fibronectin reverse (5′-CCTCGGTGTTGTAAGGTGGA-3′), peroxisome proliferator-activated receptor-γ (PPAR-γ) forward (5′-GCGGAGATCTCCAGTGATATC-3′), and reverse (5′-TCAGCGACTGGGACTTTTCT-3′), transforming growth factor-β (TGF-β) forward (5′-GAGCAACATGTGGAACTCTA-3′), TGF-β reverse (5′-TGAATCGAAAGCCCTGTATT-3′), vascular endothelial growth factor (VEGF) forward (5′-ACCCAACAACCACCTATGCT-3′), reverse (5′-TGCACTCATTGGTGGAGGTA-3′), GAPDH forward (5′-GTGCCAGCCTCGTCTCATAG-3′), GAPDH reverse (5′-GAACTTGCCGTGGGTAGAGTC-3′).

### Punch biopsy from human tissue

A single localized scleroderma patient consented to undergo punch biopsy, which was conducted prior to and 12 months after treatment. The final biopsy was performed next to the initial biopsy position. The obtained tissue was stained by H&E and MT staining, and immunohistochemical analysis was performed with perilipin. Photographs of the treatment site were also obtained using the same digital camera and a VISIA imager.

### Statistical analyses

Data are expressed as means ± standard deviation (SD). The results were analyzed by the independent Student’s *t* test (two groups at a single timepoint) and one-way analysis of variance (more than two groups at a single timepoint) using SPSS, version 21 (IBM Corporation, Armonk, NY, USA). A value of *p* < 0.05 was set for statistical significance.

## Results

### Fat grafting alleviates sclerotic changes and induces subcutaneous and cutaneous adipocyte accumulation in both human and mice

**Fig. 1:**
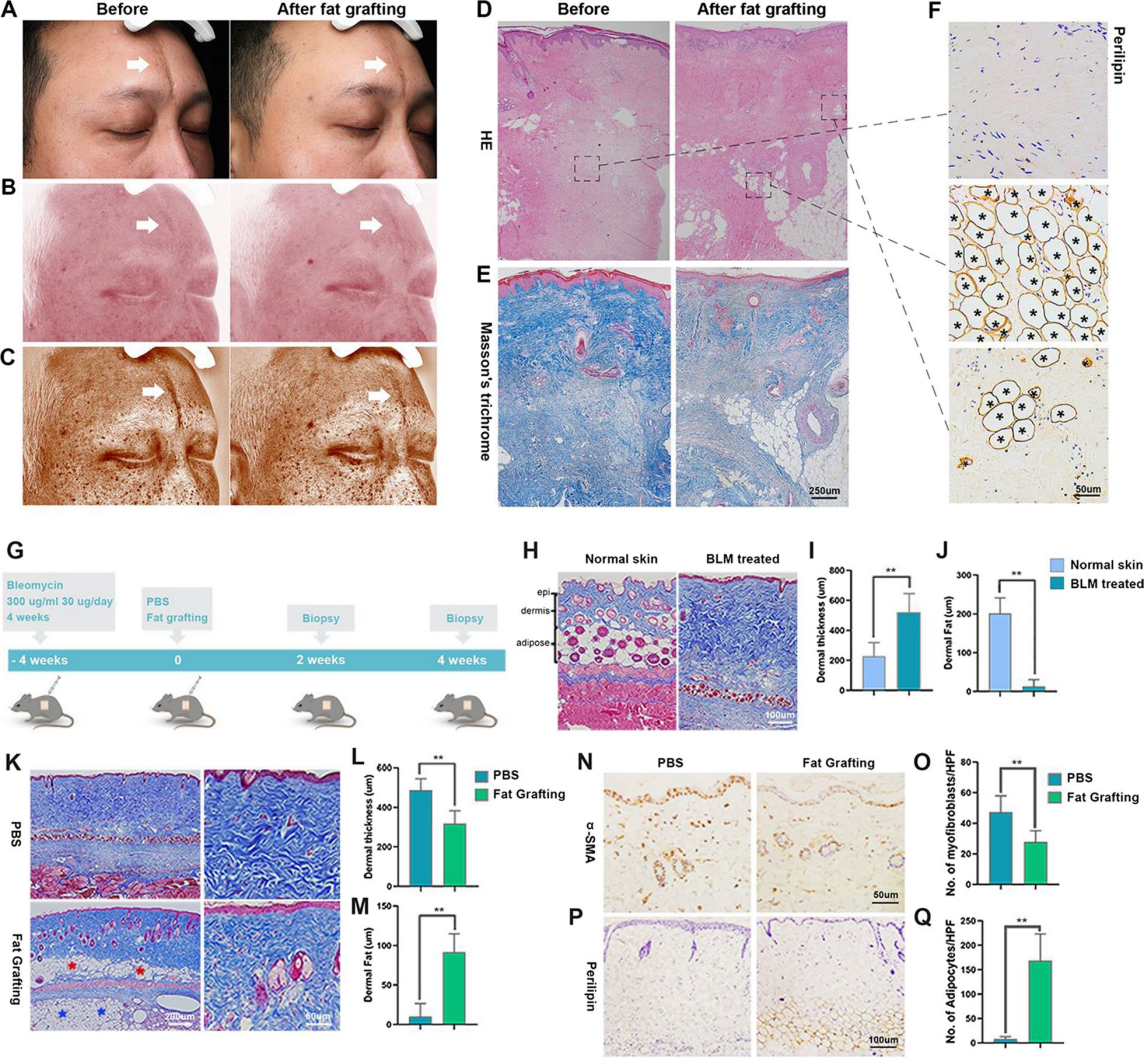
Histologic analysis of fibrotic skin after fat grafting in human and mouse tissues. **A** Localized scleroderma (white arrows) on the patient’s left forehead before and after fat grafting for one year. **B** Increased vascularization (white arrows) in the skin lesion after fat grafting. **C** VISIA images revealed even complexion after grafting. **D**, **E** Skin biopsy before and one year after fat grafting. **F** Subcutaneous and cutaneous adipocyte accumulation (black asterisks) after fat grafting in the patient. **G** Epididymal fat pads (200 μL) from AdipoqCreER^+^;mT/mG transgenic mice were transferred beneath the skin lesion of the BLM-treated mice. **H** MT staining of normal or BLM- treated C57 skin. Scale bar = 100 μm. Measurement of **I** dermal thickness and **J** dermal fat. **K** Masson staining of the samples from BLM-treated mice receiving fat graft or PBS treatment. Measurement of **L** dermal thickness and **M** dermal fat. **N** Immunohistochemistry of α-SMA+ in the PBS or fat grafting group. **O** Semiquantitative analysis of α-SMA+ cells in the skin lesion. **P** Immunohistochemistry of perilipin in the PBS or fat grafting group. **Q** Semiquantitative analysis of perilipin+ cells in the skin lesion. (**p < 0.01, *n* = 5)

Twelve months after undergoing fat grafting, the appearances of lesions in a patient were significantly improved (Fig. 1A). VISIA imaging showed increased vascularization (Fig. 1B) and decreased pigmentation (Fig. 1C). Posttreatment biopsy showed loosened collagen and adipocyte accumulation within the dermal layer (Fig. 1D, E). Immunohistochemical analysis of perilipin staining showed that few positive cells were found before treatment. Twelve months after fat grafting, the surviving transplanted fat were strongly positive for perilipin staining and scattered perilipin+ adipocytes were found in the dermal layer (Fig. 1F).

To verify the findings observed in humans, we used a bleomycin-induced mouse scleroderma model for in vivo analysis (Fig. 1G). After the subcutaneous injection of BLM for 4 weeks, it was clear that the mice developed sclerosis with significantly thickened skin and decreased dermal fat thickness (Fig. 1H–J). Four weeks after fat grafting, the skin lesion showed loosening of collagen fiber, reduced dermal thickness, and re-accumulation of dermal fat (Fig. 1K–M). The number of α-SMA+ cells was significantly decreased in the fat grafting group (Fig. 1N, O). Moreover, increasing numbers of perilipin+ adipocytes accumulated in the skin lesions (Fig. 1P, Q).

### Fat graft-derived cells appeared in the skin lesion

**Fig. 2:**
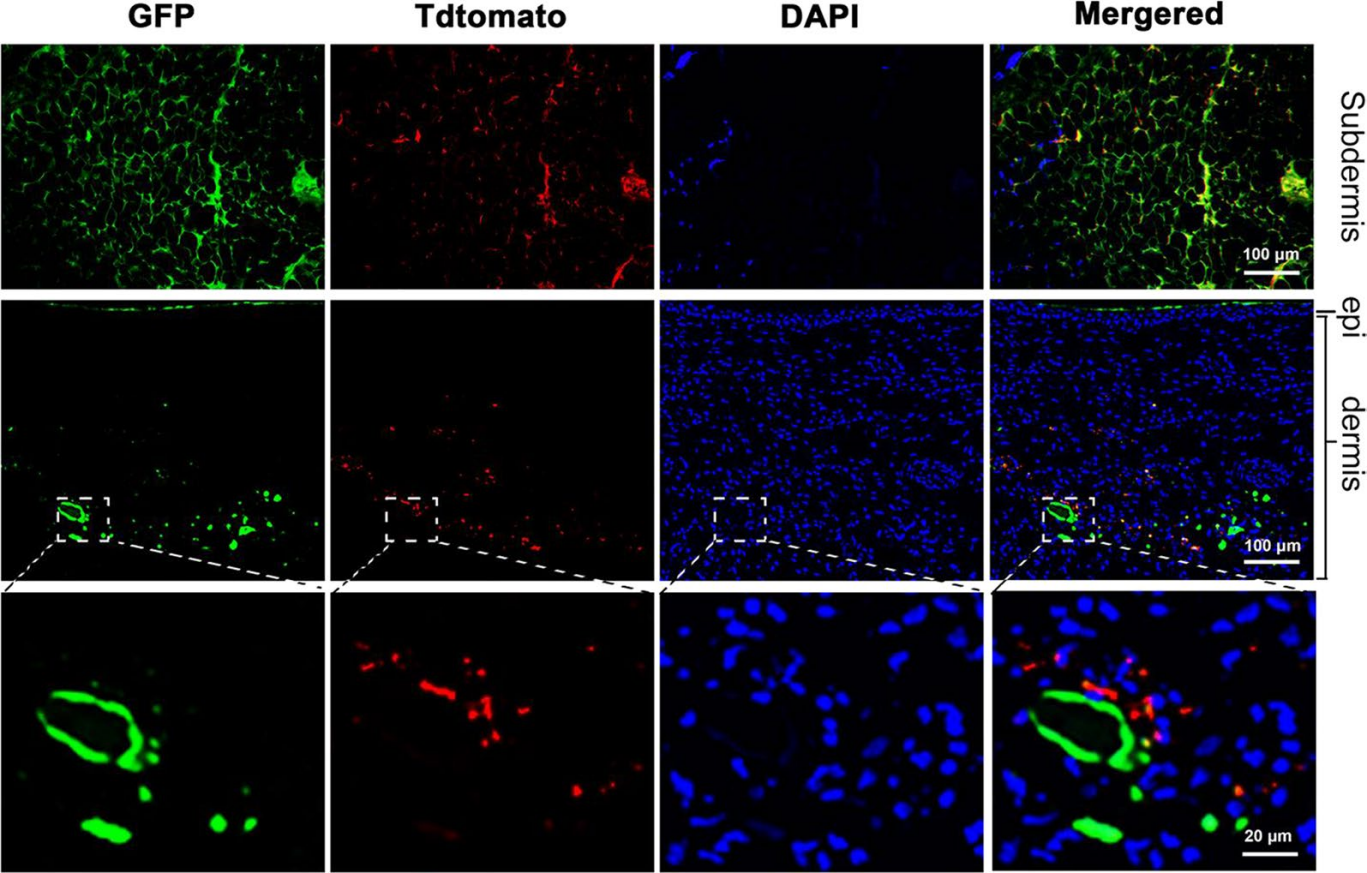
Cell trace from transplanted fat in the skin lesion. Immunofluorescent staining of GFP and Tdtomato. (Upper panel) Representative images of the transplanted fat under the dermis (upper panel; scale bar = 100 μm), fluorescent cells within the dermis (middle panel; scale bar = 100 μm), and fluorescent cells in the dermis at high magnification (lower panel; scale bar = 20 μm)

To trace the adipocytes from fat grafts, subcutaneous WAT was harvested from AdipoqCreER^+^;mT/mG mice after tamoxifen activation and transferred to the skin lesion in BLM-treated wild-type C57 mice. Two weeks after transplantation, the transferred fat was detected in the subcutaneous layer (Fig. 2, the upper layer). Surprisingly, we discovered that SVF-derived (Tdtomato-positive) and adipocyte-derived (GFP-positive) cells were found in the skin lesion, and some of the GFP+ cells did not have a typical adipocyte morphology, but instead were spindle shaped (Fig. 2, the middle and lower layers).

### Adipocytes from fat grafts undergo dedifferentiation and redifferentiation

**Fig. 3:**
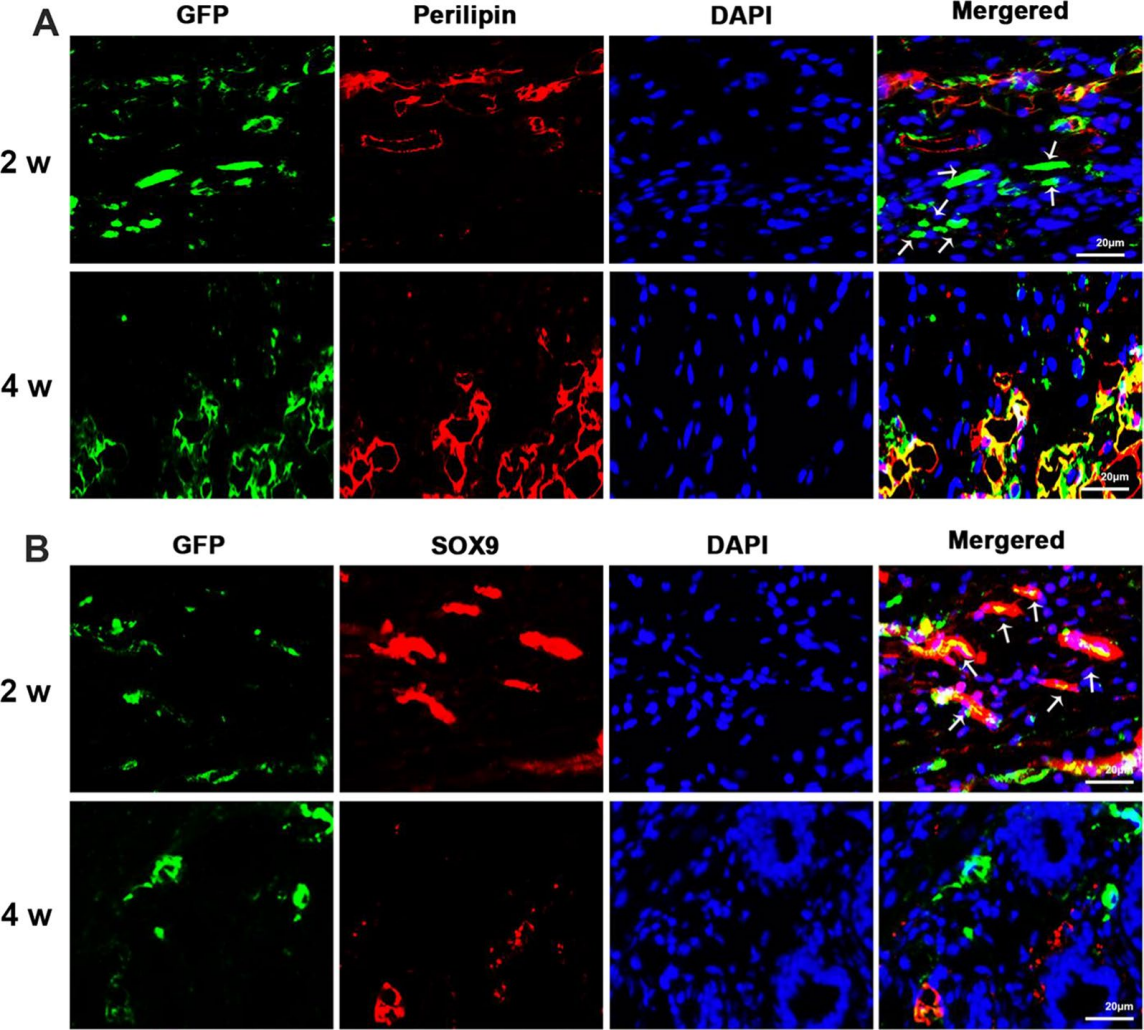
Cell type identification of fluorescent cells in the skin lesion. **A** Immunofluorescent staining of skin lesion samples from mice receiving fat grafts of AdipoqCreER^+^;mT/mG mice with GFP and perilipin. Scale bar = 20 μm. **B** Immunofluorescent staining of skin lesion samples from mice receiving fat grafts of AdipoqCreER^+^;mT/mG mice with GFP and Sox9. Scale bar = 20 μm

It has been reported that mature dermal adipocytes undergo dedifferentiation and redifferentiation in response to certain stimuli [20]. To identify the cell type of GFP+ cells and determine whether dedifferentiation of adipocytes occurred in the skin lesion, perilipin and Sox9 were used to indicate the presence of adipocytes and dedifferentiated adipocytes, respectively. At 2 weeks after grafting, some of the GFP+ cells were co-localized with perilipin, but the others were not; however, at 4 weeks, an increasing number of GFP+ cells were positive for perilipin (Fig. 3A). On the other hand, at 2 weeks, some of the GFP+ cells were positive for Sox9; however, at 4 weeks, the co-localization of GFP and Sox9 was decreased (Fig. 3B). These results suggest that adipocytes from the transferred fat underwent dedifferentiation and redifferentiation in the skin lesion.

### Adipose-derived cells reconstructed adipose layer and reversed skin fibrosis

**Fig. 4:**
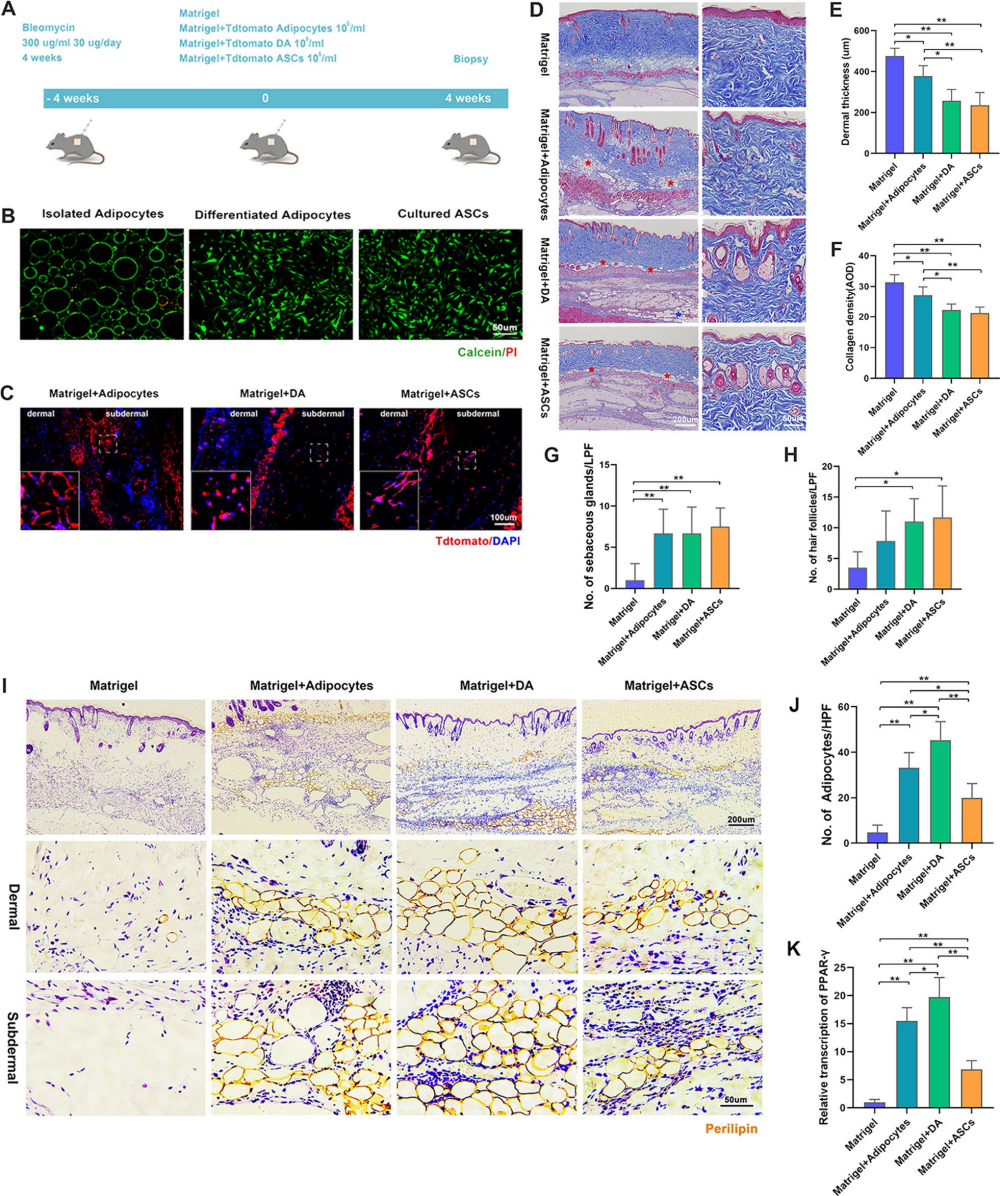
Assessment of the therapeutic effect of adipose-derived cells on BLM-induced fibrotic skin. **A** Study design of transplantation with adipose-derived cells for the treatment of fibrotic skin in mice. **B** Before transplantation of the fluorescent cells into the mice, they were stained with Calcein/PI to assess cellular viability. **C** Tdtomato immunofluorescent staining of skin lesion after transplantation in vivo. **D** MT staining of skin lesion. **E** Measurement of dermal thickness. **F** Semi-quantitative analysis of collagen density. **G** Semi-quantification of the number of sebaceous glands. **H** Semi-quantification of the number of hair follicles. **I** Perilipin staining of skin lesion after transplantation in vivo showed adipocyte accumulation in the skin. **J** Semi-quantification of the number of adipocytes. **K** qRT-PCR analysis of PPAR-γ transcription showed enhanced adipogenesis in DA and adipocyte groups (*p < 0.05; **p < 0.01, *n* = 5)

To evaluate the therapeutic potential of the different adipose-derived cells on scleroderma lesions, we transplanted fluorescent Tdtomato+ adipocytes, DAs, and ADSCs combined with Matrigel into the BLM-treated wild-type C57 mice (Fig. 4A). The cells used for transplantation were mostly positive for Calcein, which indicated that most cells were alive (Fig. 4B). After transplantation in vivo for 4 weeks, fluorescent cells were detected in the subdermal and dermal layers (Fig. 4C). Masson's trichrome (MT) staining was performed to evaluate the therapeutic effects of transplantation (Fig. 4D). Dermal thickness (Fig. 4E) and collagen density (Fig. 4F) were significantly reduced in the DA- and ASC-injected groups and in the adipocyte-injected group. The number of hair follicles and sebaceous glands in the dermis were significantly higher in the DA- and ASC-injected groups than that in the Matrigel group (Fig. 4G, H). Adipocytes accumulated in the subdermal and dermal layers of skin lesions from the DA-, adipocyte-, and ASC-injected groups, but not in the Matrigel group (Fig. 4I, J). PPAR-γ transcription was significantly increased in the adipocyte-, DA-, and ASC-injected groups, with the DA group showing the highest expression (Fig. 4K).

### Adipose-derived cells decreased myofibroblast accumulation and improved angiogenesis in skin lesions

**Fig. 5:**
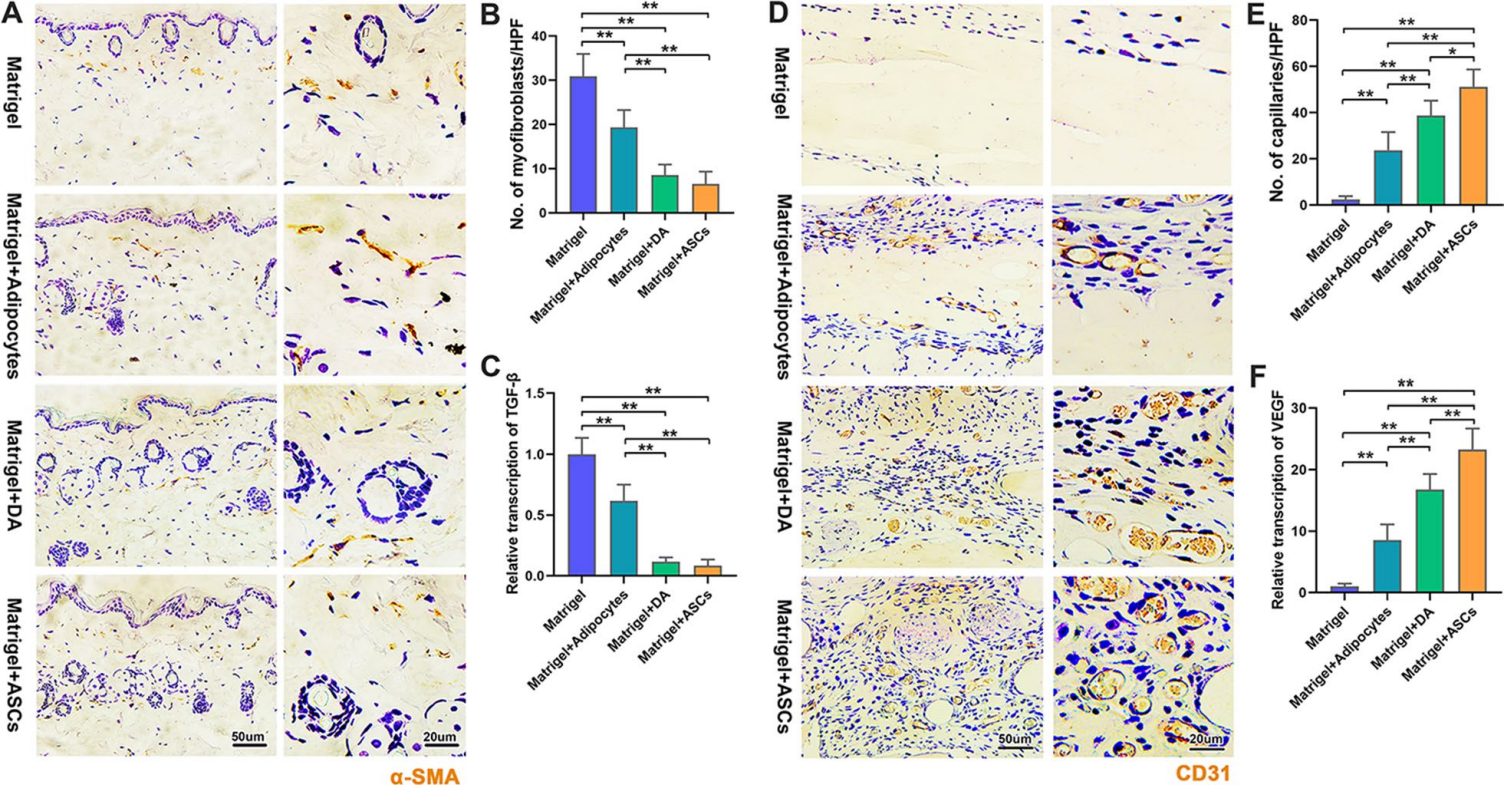
Myofibroblast accumulation and angiogenesis level among different treatment groups. **A** Immunochemistry with α-SMA of skin lesions in different treatment groups. **B** Semi-quantitative analysis of the number of α-SMA+ myofibroblasts in the dermis. **C** qRT-PCR analysis of TGF-β expression in skin lesions of different treatment groups. **D** Immunochemistry with CD31 of skin lesions in different treatment groups. **E** Semi-quantitative analysis of the number of CD31+ cells. **F** qRT-PCR analysis of VEGF-α expression. (*p < 0.05; **p < 0.01, *n* = 5)

The relative abundance of myofibroblasts in skin lesions differed among groups. The number of myofibroblasts in the dermis was highest in the Matrigel group and lowest in the DA- and ASC-injected groups (Fig. 5A, B). TGF-β expression was significantly decreased in the adipocyte-, DA-, and ASC-injected groups (Fig. 5C).

Immunochemistry analysis using CD31 was performed to identify endothelial cells in the skin lesions (Fig. 5D). Some cell infiltration and almost no CD31+ cells were found in the Matrigel group. The number of CD31+ cells was highest in the ASC group (Fig. 5E). VEGF expression was significantly increased in the adipocyte-, DA-, and ASC-injected groups (Fig. 5F).

### Adipose-derived cells ameliorated dermal inflammation

**Fig. 6:**
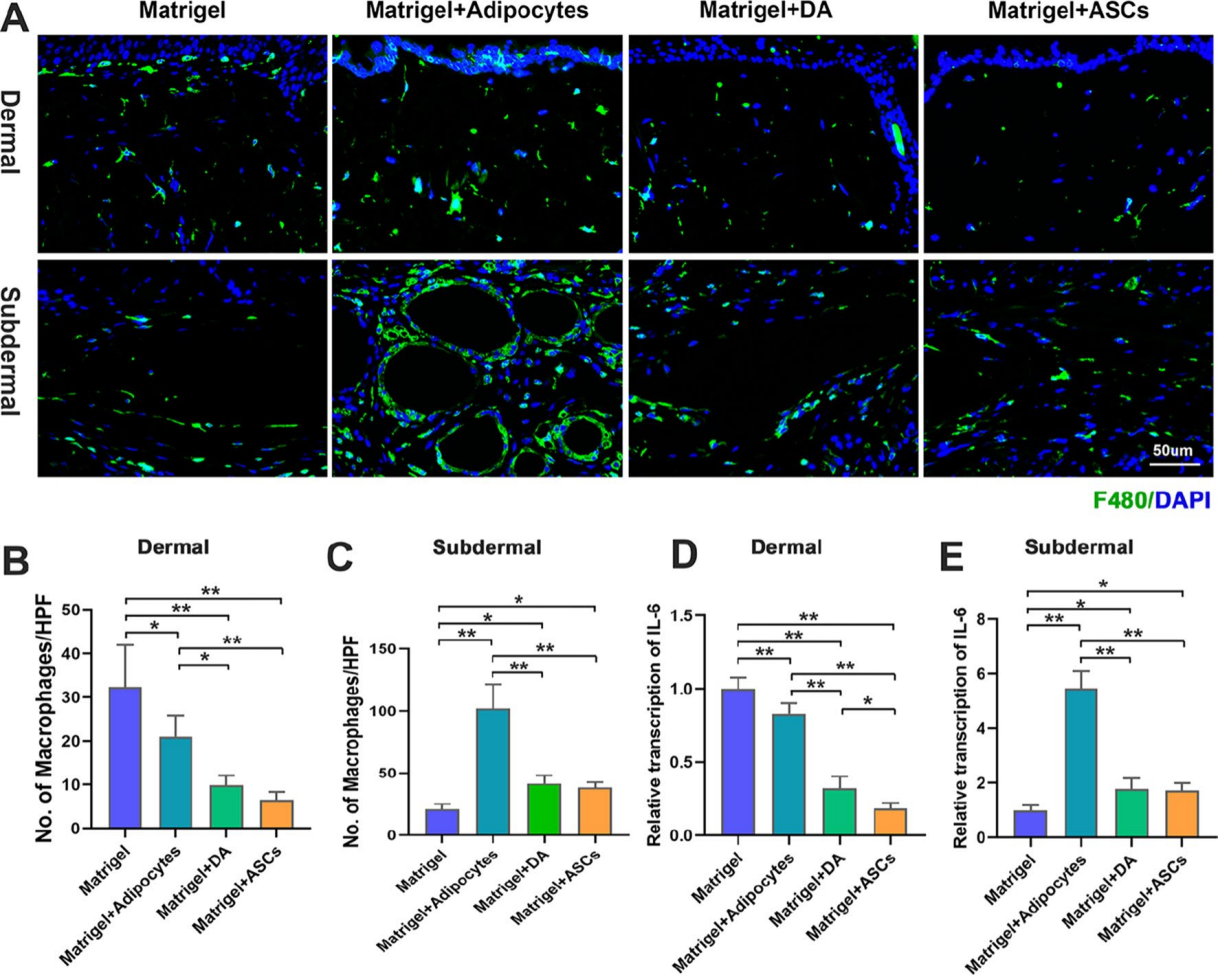
Subdermal and dermal inflammation levels among different treatment groups. **A** F4/80 immunofluorescent staining of skin lesions in different treatment groups. Semi-quantification of the F4/80+ cells in the **B** dermal and **C** subdermal layers. qRT-PCR analysis of IL-6 transcription in the **D** dermal and **E** subdermal layers. (*p < 0.05; **p < 0.01, *n* = 5)

To assess the degree of inflammation after treatment, we evaluated the levels of macrophage infiltration and pro-inflammatory cytokine transcription. F4/80+ macrophages were found in the subdermal and dermal layers of each group. The crown-like structure formed by F4/80+ cells, which indicates macrophages surrounding dead adipocytes, was found in the cells of the adipocyte group, while the other groups had scattered F4/80+ cells with no crown-like morphology (Fig. 6A). The dermal layers of the adipocyte, DA, and ASC groups had lower macrophage infiltration than that of the Matrigel group (Fig. 6B). However, in the subdermal layer, the adipocyte group showed the highest macrophage infiltration, while the Matrigel group had the lowest (Fig. 6C). IL-6 transcription level showed a trend similar to that of macrophage infiltration; in the dermal layer, IL-6 transcription was significantly lower after the injection of adipocyte, DA, and ASC (Fig. 6D). In terms of the subdermal layer, the Matrigel group showed the lowest IL-6 transcription, while the adipocyte group had the highest (Fig. 6E).

### ASCs, DAs and adipocytes inhibited ECM gene expression in co‐cultured SFs

**Fig. 7:**
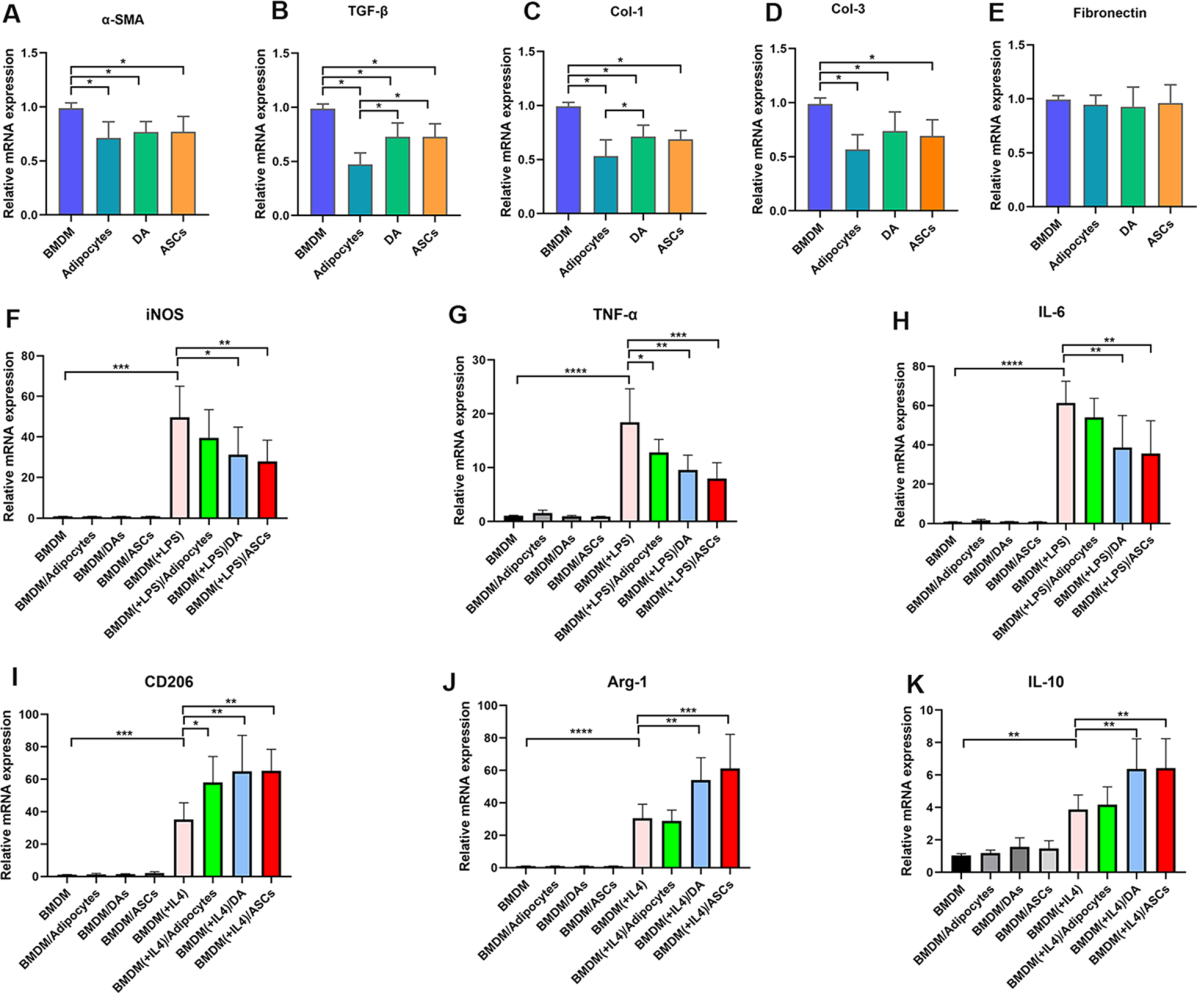
The effect of ASCs, DAs and adipocytes on scleroderma fibroblasts and M1/M2 macrophage polarization. Quantitative PCR analysis of various gene expressions in human scleroderma fibroblasts (hSF) without or with co‐culture of ASCs, DAs or adipocytes, respectively, for α‐SMA (**A**), TGF-β1 (**B**), collagen I (**C**), collagen III (**D**) and fibronectin (**E**). mRNA expression levels of iNOS (**F**), TNF-α (**G**) and IL-6 (**H**) in BMDMs, which were treated with or without LPS and were co-cultured with or without ASCs, DAs or adipocytes. mRNA expression levels of CD206 (**I**), Arg-1 (**J**), and IL-10 (**K**) in BMDMs, which were treated with or without IL-4 and were co-cultured with or without ASCs, DAs or adipocytes (*p < 0.05; **p < 0.01; ***p < 0.001; ****p < 0.0001, *n* = 5)

After co-culture with ASCs, DAs and adipocytes, respectively, α-SMA, a marker for myofibroblasts, was also significantly inhibited for its gene expression in the co-culture groups (*p* < 0.05, Fig. 7A). Moreover, the expression level of pro-fibrotic gene, TGF-β was significantly reduced in the co-culture groups (*p* < 0.05, Fig. 7B). The gene expression levels of ECM molecules were inhibited for Col 1 (*p* < 0.05, Fig. 7C) and Col 3 (*p* < 0.05, Fig. 7D). No significant difference was found in Fibronectin expression (*p* > 0.05, Fig. 7E).

### Adipose-derived cells drive anti-inflammatory M2 macrophage polarization in vitro

We then explored the effect of adipose-derived cells on macrophage polarization in vitro co-culture system. Macrophage differentiation was performed with lipopolysaccharide (LPS) and IL-4, which generates LPS-dependent classically activated M1 macrophages and IL-4-dependent alternative activated M2 macrophages. M1 macrophages were characterized using iNOS/IL-6 /TNF-α expression, and M2 macrophages were characterized using Arg1/CD206/IL-10 expression. Both ASCs and DAs were able to reduce the gene expression of iNOS, IL-6 and TNF-α expression after activation of macrophages by LPS, while adipocyets only suppressed the expression of TNF-α (*p* < 0.05, Fig. 7F–H). Furthermore, M2 macrophages co-cultured with ASCs and DAs showing the higher level of Arg1, CD206 and IL-10, while adipocytes only increased the expression of CD 206 (*p* < 0.05, Fig. 7I–K).

## Discussion

Fat grafting has shown great therapeutic effects in treating scleroderma, not only for volume loss correction, but also for skin texture improvement [23]. During clinical practice, we also noticed skin texture improvement in patients receiving fat grafts, including softened skin, improved vascularization, and decreased pigmentation. Surprisingly, when we observed the histologic sample of the human skin lesion treated with fat grafts and compared it with pre-operation lesions, we found not only the surviving transferred fat beneath the skin lesion, but also some small clusters of adipocytes in the dermal layer. Additionally, we used a BLM-induced skin fibrosis animal model to further confirm this finding. After fat grafting in BLM-treated mice, adipocyte accumulation was observed in the subcutaneous (beneath the p.c.) and dermal layers (above the p.c.). Fluorescent staining results suggested that the subcutaneous fat layer contained transferred fat with GFP-positive adipocytes and Tdtomato-positive SVF cells. Interestingly, a few fluorescent cells were found in the dermal fat layer, indicating that some adipocytes were regenerated from the host and some were derived from the transferred fat. Thus, it was confirmed that the transferred adipose tissue not only reconstructed the subcutaneous fat layer, but also participated in dermal fat regeneration. In this way, fat grating may be a better option than stem cell injection for scleroderma treatment, especially for the subtype with deep soft tissue atrophy, since fat grafting is not only anti-fibrotic but also efficient for tissue volume restoration.

In our fat grafting model, we noticed that most dermal fluorescent cells were GFP-positive, rather than Tdtomato-positive, indicating that most fat graft-derived cells in the dermal layer were adipocyte-derived. Many studies have shown that dWAT has remarkable functional pleiotropy and phenotypic plasticity. However, few studies have reported the cellular plasticity of adipocytes in subcutaneous white adipose tissue or transferred fat. Our study showed for the first time that GFP-positive adipocytes from the transferred fat of AdipoqCreER^+^;mT/mG mice appeared in the skin lesions of BLM-treated wild-type C57 mice, which might caused by direct injection to the dermal layer or migration of adipocytes from subcutaeous transffered fat. It has been reported that after wounding, dermal adipocytes tend to change their fate and trans-differentiate near the wound area using an unusual adhesion-independent, actomyosin-driven, peristaltic mode of motility to contribute to wound healing. However, if the adipocytes could migrate to the skin lesion, it is unclear what attracts adipocytes to migrate to the target region. Inflammatory cytokines or unique signal proteins may be responsible for attraction of adipocytes [21]. Further investigations are required to elucidate the adipocyte identity and behavior in conditions with skin fibrosis. In dermal fibrosis, adiponectin-positive intradermal progenitors give rise to dermal myofibroblasts [24]. Moreover, adipocytes in dWAT undergo dedifferentiation and redifferentiation during the hair growth cycle [25]. In our study, similar results were found in that some of the GFP-positive cells were not round and adipocyte-shaped, but spindle-shaped. Zhang et al. suggested that adipocytes undergo dedifferentiation and redifferentiation under physiological and pathophysiological conditions [26]. Thus, we hypothesized that spindle-shaped GFP-positive cells in the dermal layer of skin lesions might be dedifferentiated adipocytes and used Sox-9 and perilipin to identify the phenotype of the cells. The phenotype and cellular properties of dedifferentiated adipocyte-derived progeny cells are still unclear, but various recent studies on the surface markers of DAs have shown that they are CD29, CD44, CD73, CD90, CD105, RUNX2, SPP1, SP7, BGLAP, PTH1R and SOX9 positive and CD14, CD34, CD45, CD117, CD133, CD271, CD309, HLA-DR negative; these surface markers are nearly identical to those of ADSCs [27, 28]. Since there are no identified specific markers for DAs, we selected Sox9 as a representative marker for DAs. Thus, GFP-positive cells which also express the SOX9 surface marker would be classified as DAs. Immunofluorescent staining showed that at 2 weeks, a number of GFP-positive cells were Sox9-positive and perilipin-negative, indicating the presence of DAs. At 4 weeks, increasing number of GFP-positive cells were perilipin-positive, while the number of Sox9+ /GFP+ cells were decreasing. These results suggest that the adipocytes in transferred fat migrate to the skin lesion and undergo dedifferentiation; after staying in the skin lesion, DAs redifferentiate into adipocytes.

Though we discovered the presence of adipocytes or DAs in scleroderma-associated skin lesions after fat grafting, their therapeutic effect on the lesion was unclear. We injected adipocytes and DAs combined with Matrigel into the skin lesions and compared them with ASCs-treated lesions. Treatment with ASCs, DAs, and adipocytes reduced dermal thickness, decreased levels of collagen, and increased the levels of vessels in BLM-induced sclerotic skin. Macrophages contributed to the onset and progress of scleroderma [29]. Macrophage infiltration IL-6 levels in skin lesions were significantly reduced in the ASC, DA, and adipocyte groups compared to those in the Matrigel group. Notably, inflammation levels in the subcutaneous layer, where cells were injected, were higher in the adipocyte group than those in the other groups. This finding might be related to the apoptosis of adipocytes after transplantation. It has been reported that, compared with ASCs, adipocytes are fragile and can hardly survive under ischemic conditions, resulting in cell death and subsequent inflammation [30–32]. This large-scale cell death and high level of inflammation compromises the therapeutic effect of adipocytes. Thus, injection of adipocytes could reverse cutaneous fibrosis, but the anti-fibrotic result cannot be compared with those of ASCs or DAs.

DAs are not only multipotent, but also pluripotent. They have the potential to differentiate to any of the three germ layers (endoderm, mesoderm, and/or ectoderm) and can develop into adipose cells, osteoblasts, chondrocytes, skeletal myocytes, smooth muscle cells, cardiomyocytes, endothelial cells, and neuronal cells [33–36]. DAs showed impressing therapeutic effects in different conditions requiring tissue regeneration, including bone defects, intervertebral disc degeneration, infarcted myocardium, and hindlimb ischemia. [37–40]. Since we discovered that adipocytes in transferred fat migrate to skin lesions and undergo dedifferentiation, we assessed the therapeutic effect of DAs on an animal skin fibrosis model. DAs improved angiogenesis and adipogenesis and ameliorated macrophage infiltration in the dermal layer of skin lesion, which led to reversal of dermal fibrosis. Compared with ASCs and adipocytes, DAs showed the strongest adipogenic capacity. This could be attributed to the fact that DAs are derived from adipocytes, so they have a stronger tendency than ASCs for adipogenic differentiation and promoting adipogenesis in dermis. Compared with adipocytes, these cells have higher hypoxia tolerance, so they survive better after transplantation and can considerably contribute to cell function. Moreover, if adipocytes need to undergo dedifferentiation to perform their function, dedifferentiated cells can directly skip this step and play a variety of roles, thus exhibiting better therapeutic effect than adipocytes.

## Conclusion

In this study, we demonstrated the direct participation of adipocytes from fat grafts in skin repair for scleroderma and evaluated the role of different adipose-derived cells. This is the first study providing visible evidence that the cell component from adipose tissue appeared in the skin lesions. The adipocytes underwent dedifferentiation and redifferentiation in the dermal layer and participated in the adipocyte accumulation in this layer. Moreover, direct use of DAs significantly improved dermal adipogenesis and reversed dermal fibrosis.

## Abbreviations

ASCs: Adipose-derived stem cells
DAs: Dedifferentiated adipocytes
ECM: Extracellular matrix
dWAT: Dermal white adipose tissue
sWAT: Subcutaneous WAT
BLM: Bleomycin
PBS: Phosphate-buffered saline
SVF: Stromal vascular fraction
H&E: Hematoxylin and eosin
MT: Masson’s trichrome
α-SMA: Alpha smooth muscle actin
SF: Scleroderma fibroblast
BMDM: Bone marrow-derived macrophages
LPS: Lipopolysaccharide
GM-CSF: Granulocyte-macrophage colony-stimulating factor
qRT-PCR: Quantitative reverse transcription-polymerase chain reaction
SD: Standard deviation
GFP: Green fluorescence protein
TNF-α: Tumor necrosis factor-α
iNOS: Inducible nitric oxide synthase
IL-6: Interleukin 6
IL-10: Interleukin-10
PPARγ: Peroxisome proliferator-activated receptor-γ
VEGF: Vascular endothelial growth factor
TGF-β: Transforming growth factor-β
